# Vibrio cholerae RND efflux systems: mediators of stress responses, colonization and pathogenesis

**DOI:** 10.3389/fcimb.2023.1203487

**Published:** 2023-05-15

**Authors:** X. Renee Bina, James E. Bina

**Affiliations:** Department of Microbiology and Molecular Genetics, University of Pittsburgh School of Medicine, Pittsburgh, PA, United States

**Keywords:** *Vibrio cholerae*, pathogenesis, efflux, adaptation, virulence

## Abstract

Resistance Nodulation Division (RND) efflux systems are ubiquitous transporters in gram-negative bacteria that provide protection against antimicrobial agents and thereby enhance survival in virtually all environments these prokaryotes inhabit. *Vibrio cholerae* is a dual lifestyle enteric pathogen that spends much of its existence in aquatic environments. An unwitting encounter with a human host can lead to *V. cholerae* intestinal colonization by strains that encode cholera toxin and toxin co-regulated pilus virulence factors leading to potentially fatal cholera diarrhea and dissemination in the environment. Adaptive response mechanisms to host factors encountered by these pathogens are therefore critical both to engage survival mechanisms such as RND-mediated transporters and to induce timely expression of virulence factors. Sensing of cues encountered in the host may therefore activate more than protective responses such as efflux systems, but also be coordinated to initiate expression of virulence factors. This review summarizes recent advances that contribute towards the understanding of RND efflux physiological functions and how the transport systems interface with the regulation of virulence factor production in *V. cholerae*.

## *Vibrio cholerae* is an enteric pathogen and the etiological agent of pandemic cholera

*Vibrio cholerae* is a comma-shaped, gram-negative bacterium that causes the acute diarrheal disease cholera. Cholera is an enteric infection characterized by a rapid onset of profuse watery diarrhea that can quickly lead to dehydration and death if left untreated (Phillips, 1968). Seven cholera pandemics have been documented since 1817 with the seventh and ongoing pandemic beginning in 1961 [reviewed by (Kaper et al., 1995)]. Currently, ~1.3 billion people are at risk for cholera with a global burden of ~2.9 million cholera cases and 95,000 deaths annually (Ali et al., 2015).

*V. cholerae* inhabits aquatic ecosystems including fresh (Bina et al., 2020; Daboul et al., 2020) and brackish water (Chakraborty et al., 1997) where these bacteria associate with aquatic invertebrates and hydrolyze chitin (Huq et al., 1983; Meibom et al., 2004). Humans naturally acquire cholera from consumption of contaminated food or water. Following ingestion, *V. cholerae* traverses the stomach and colonizes the crypts of the small intestine where cholera toxin (CT) production and activity leads to the majority of diarrhea induced in the host (Herrington et al., 1988). *V. cholerae* adaptive responses to cues encountered in the human gastrointestinal tract elicit transcriptome reprogramming by activating environmental sensing systems including two-component regulatory systems (TCS), the key virulence regulator ToxR, and other regulatory proteins [reviewed by (Childers and Klose, 2007)]. Numerous TCS have been documented to contribute towards *V. cholerae* survival in the host. For example, the Cad system mediates acid tolerance (Merrell and Camilli, 1999; Merrell and Camilli, 2000), CarRS contributes to biofilm production and antimicrobial peptide resistance (Bilecen and Yildiz, 2009; Herrera et al., 2014; Bilecen et al., 2015), the PhoBR system regulates phosphate uptake and metabolism (Pratt et al., 2010), LuxO acts as a quorum-sensing regulator (Zhu et al., 2002), the VarSA system responds to environmental pH and salt (Jang et al., 2011), the VieSA system responds to the second messenger cyclic diguanylate (Tischler and Camilli, 2005; Martinez-Wilson et al., 2008), FlrBC is a flagellar regulatory protein (Correa et al., 2000), the VxrAB system senses cell-wall stress and regulates type VI secretion (Cheng et al., 2015; Teschler et al., 2017; Peschek et al., 2020), and the osmoregulator OmpR-EnvZ system modulates virulence, adaptation to alkaline pH and responses to membrane intercalating agents (Kunkle et al., 2019; Kunkle et al., 2020a; Kunkle et al., 2020b). In many of these systems the specific environmental cues that stimulate their activity are poorly understood.

**Figure 1:**
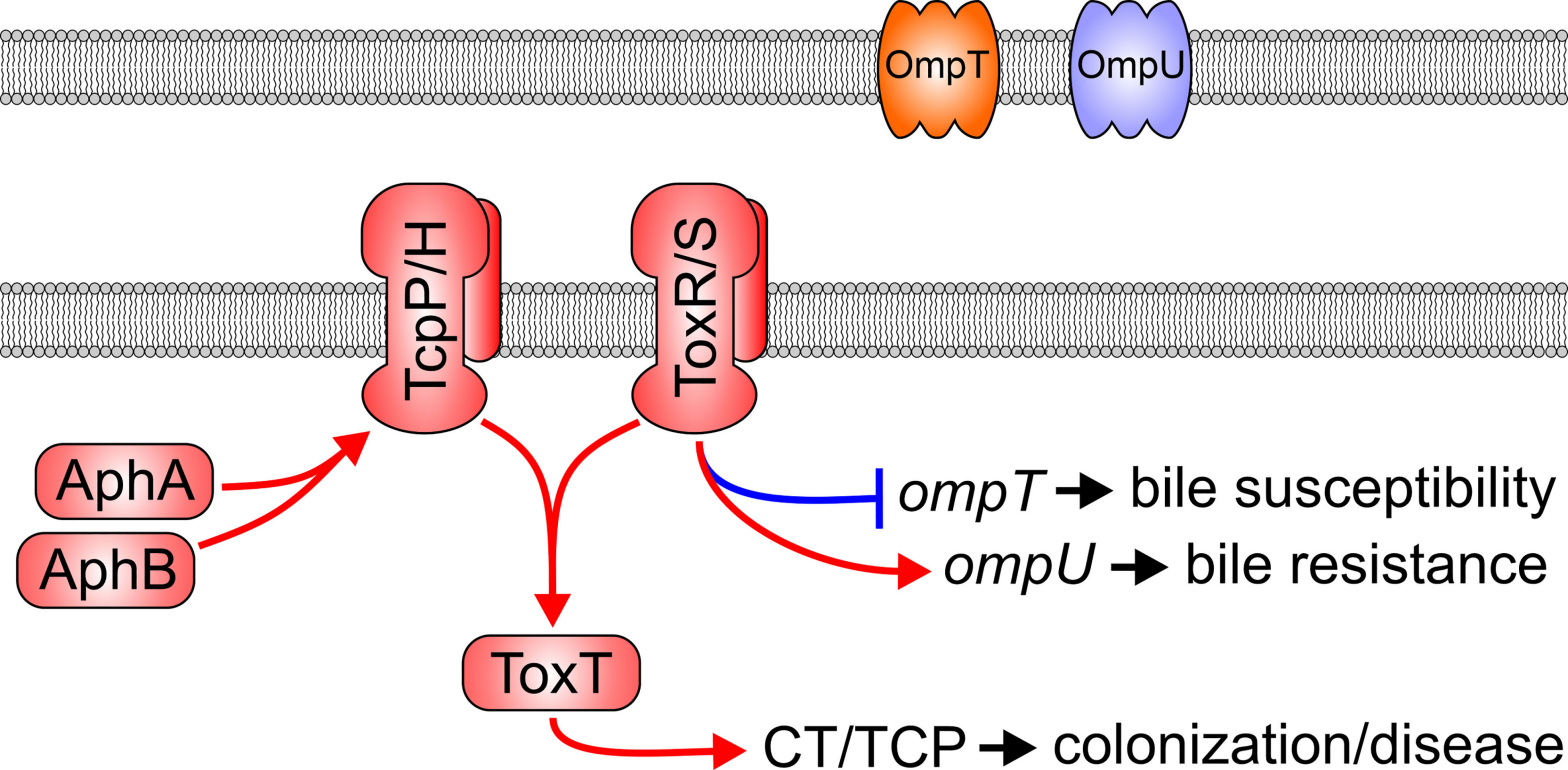
The *Vibrio cholerae* ToxR virulence regulon. In response to low cell density and low oxygen tension, the AphA and AphB transcription factors bind to the promoter and activate transcription of the *tcpPH* operon. TcpP/H and ToxR/S then bind to the *toxT* promoter to induce its transcription. ToxT then activates the transcription of multiple virulence genes including the genes encoding for production of cholera toxin (CT) and the toxin co-regulated pilus (TCP). ToxR/S, independently of TcpP/H, also modulates transcription of outer membrane porins OmpU and OmpT.

*V. cholerae* must negotiate a range of contrasting physiological environments to reach the site of colonization in the small intestine; the ability to coordinate the expression of virulence genes in response to environmental cues is therefore critical to cholera pathogenesis. This process is orchestrated by a hierarchical regulatory pathway called the ToxR virulence regulon which consists of five major regulatory proteins: AphA, AphB, TcpP, ToxR, and ToxT (**Figure 1**) (Childers and Klose). The regulon is induced by AphA and AphB binding to the promoter and initiating transcription of *tcpPH* (Kovacikova and Skorupski, 1999; Skorupski and Taylor, 1999). ToxR’s ancestral role is to modulate expression of outer membrane porins OmpU and OmpT (Provenzano and Klose, 2000; Provenzano et al., 2001), but together with TcpP, ToxR induces transcription of *toxT* (Hase and Mekalanos, 1998). ToxT then directly activates the expression of the genes encoding the two primary *V. cholerae* virulence factors, CT and the toxin coregulated pilus (TCP) (**Figure 1**) (DiRita et al., 1991). TCP is a type IV pilus required for colonization, and CT is the enterotoxin that causes the overwhelming majority of secretory diarrhea that is the hallmark of cholera and essential for *V. cholerae* epidemic spread [7]. Late during infection when *V. cholerae* reaches high cell densities in the intestinal lumen, a phenotypic shift occurs in the pathogen’s transcriptome that results in the repression of virulence and upregulation of genes involved in dissemination and transmission (Merrell et al., 2002; Bina et al., 2003; Xu et al., 2003; Larocque et al., 2005; Schild et al., 2007). Dissemination genes enhance *V. cholerae* survival in aquatic ecosystems whereas transmission genes induce a transient hyperinfectious phenotype that promotes epidemic spread (Merrell et al., 2002). Signals received by the bacteria that lead to expression of late infection genes are unknown.

Similar to TcpP and TcpH where TcpH stabilizes TcpP (Hase and Mekalanos, 1998), the virulence regulator ToxR, together with its cognate accessory protein ToxS, forms a transmembrane one-component transcription factor that responds to environmental cues and is critical to *V. cholerae* pathogenesis (Miller et al., 1987; Miller et al., 1989). The ToxR/S heterodimer consists of two proteins; the ToxR moiety spans the membrane with a DNA-binding N-terminal cytoplasmic and a C-terminal periplasmic sensing domain (PPD). The *toxR* gene is encoded upstream of its accessory *toxS* which gene product is also anchored in the membrane and is required for full ToxR activity (Miller et al., 1989). Agonists have been shown to promote interactions between ToxR and ToxS, and/or affect the oxidation state of the PPD’s cysteine residues to activate ToxR (Fengler et al., 2012; Midgett et al., 2017). Interestingly, all ToxR agonists described to date [bile salts (Provenzano et al., 2000; Ante et al., 2015b), indole (Howard et al., 2019) and cyclo(Phe-Pro) (Bina and Bina, 2010)] are substrates of the Resistance Nodulation Division (RND) efflux systems suggesting the possibility of a small molecule feedback mechanism between ToxR and RND-mediated efflux.

*V. cholerae* must overcome several barriers in transit across the gastrointestinal tract including toxic metabolites produced by the resident microbiota, bile salts and products of the innate immune system [reviewed by (Peterson and Gellings, 2018)]. *V. cholerae* transcriptome modulation enhances resistance against these harmful products by restricting outer membrane permeability and by increasing efflux system activity (Wibbenmeyer et al., 2002; Bina et al., 2008). Multiple drug efflux systems belonging to the RND superfamily are essential for intrinsic antimicrobial resistance against a wide range of compounds in most gram-negative bacteria [reviewed by (Li et al., 2015; Colclough et al., 2020)]. While RND systems contribute to antimicrobial resistance, numerous studies indicate that these transporters fulfill critical physiological functions independent of their role in antibiotic resistance [reviewed by (Piddock, 2006; Alvarez-Ortega et al., 2013)]. This review provides an update on the contributions of the RND efflux systems to *V. cholerae* host-pathogen interactions.

## RND efflux systems are ubiquitous transporters among gammaproteobacteria

RND efflux pumps were first discovered in the early 1990’s because of their contribution to antibiotic resistance (Poole et al., 1993). Subsequent studies revealed that RND efflux transporters were ubiquitous in gram-negative bacteria and that most bacteria encoded multiple and distinct RND transporters in their chromosome [reviewed by (Li et al., 2015)]. More recently, RND system gene clusters encoded on plasmids were identified in multiple antibiotic resistant pathogens enhancing the potential for their horizontal spread (Lv et al., 2020). RND transporters efflux a wide variety of structurally and chemically diverse compounds including various classes of antibiotics, antimicrobial peptides, detergents and dyes (Li et al., 2015). This sets RND systems apart from other transporters and provides intrinsic resistance to diverse antimicrobials in their respective host bacteria. Broad substrate specificity has been recognized as an important factor in the evolution of multiple drug resistance in gram-negative bacteria including pathogens identified by the Centers for Disease Control as priority health threats due to emergent antibiotic resistance (Solomon and Oliver, 2014).

**Figure 2:**
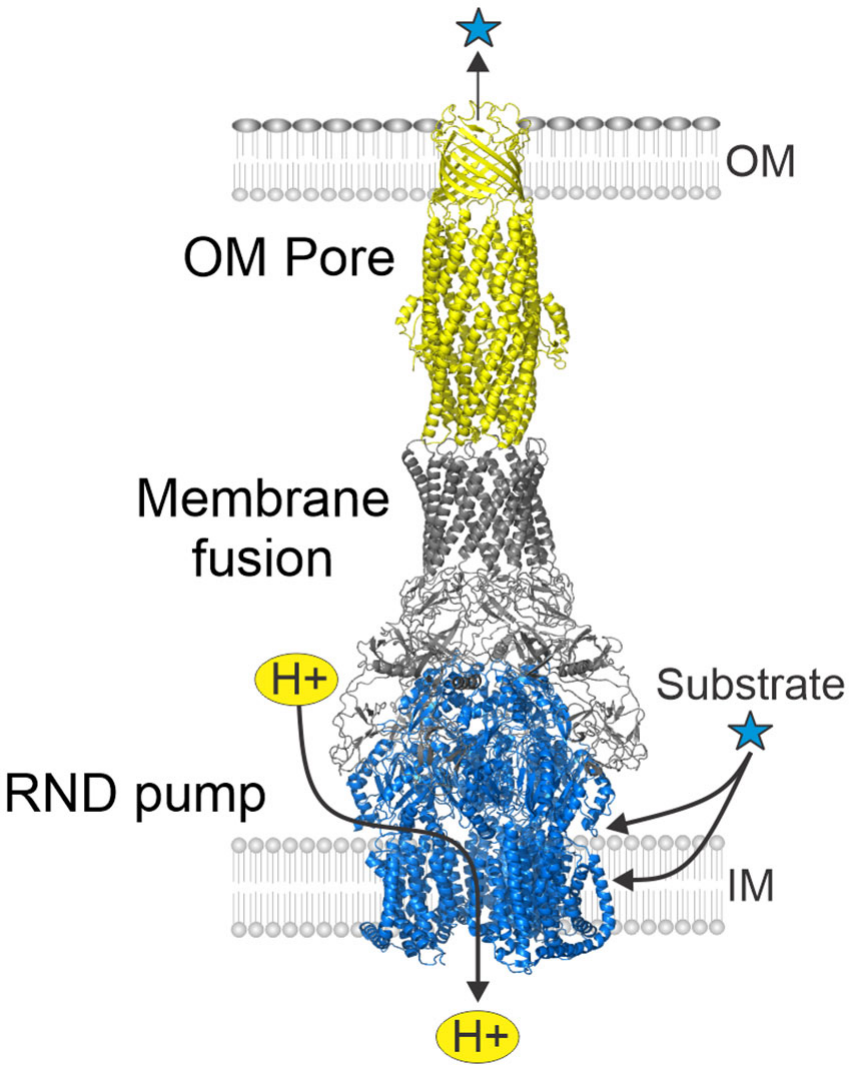
Structure of tripartite RND efflux systems. RND efflux systems consists of an outer membrane pore protein (yellow) orthologous to *E. coli* TolC, a membrane fusion protein (gray) and an inner membrane RND-family pump protein (blue). The RND systems function as substrate-proton antiporters and collect substrates from the periplasm and cytoplasmic membrane for export into the extremal milieu. Figure derived from PDB ID: 5V5S (Wang et al., 2017).

RND efflux systems comprise remarkable tripartite transporters consisting of an outer membrane pore protein ortholog of *Escherichia coli* TolC, a periplasmic adapter protein belonging to the membrane fusion protein family, and the integral inner membrane RND superfamily pump protein (Colclough et al., 2020). The transporter’s structure exhibits a 3:6:3 stoichiometry (pump to membrane fusion to pore protein peptide ratio) that forms a quaternary complex with an interior channel that spans the entire width of the cell envelope (**Figure 2**) (Daury et al., 2016). The RND pump protein features an entry portal domain at the periplasm-cytoplasmic interface thought to facilitate the passage of substrate molecules from the periplasm and the cytoplasmic membrane for efflux into the extracellular milieu. The transporter’s pump antiport activity is provided by the proton motive force that powers the efflux activity (Venter et al., 2015). Interestingly, the *V. cholerae* VexEF RND transporter requires sodium for activity; however, the underlying mechanism governing this requirement remains to be investigated (Rahman et al., 2007).

Crystallized protein domains of RND efflux systems in the presence and absence of substrates or inhibitors provided a wealth of information on assembly and function [see (Alav et al., 2021; Athar et al., 2023)]. Analyses of the crystal structures of multiple RND transporter homologues revealed that RND pump proteins are structurally conserved; they all include multiple substrate entry portals and several substrate binding sites which mediate uptake of substrates from the periplasm and from within the cytoplasmic membrane. Large substrate-binding pockets along the periplasm-cytoplasmic membrane interface appear to facilitate selection of substrates with defined physicochemical properties with specific amino acid residues determining the selectivity and specificity of the binding pocket. Collectively, multiple binding sites and substrate entry portals explain the broad substrate specificity of RND transporters. Substrate molecules are transported across the transporter to the external environment by a peristaltic pump mechanism that results from the rotation of the three RND pump components. TolC’s export channel is composed of an outer membrane-spanning β-barrel domain joined to a periplasm-spanning α-helical domain that directly interact with the membrane-fusion proteins which function as adaptors between the pore and the pump (Koronakis et al., 2000).

## RND efflux systems in *V. cholerae* are structurally related to those in other gram-negative bacteria

**Figure 3:**
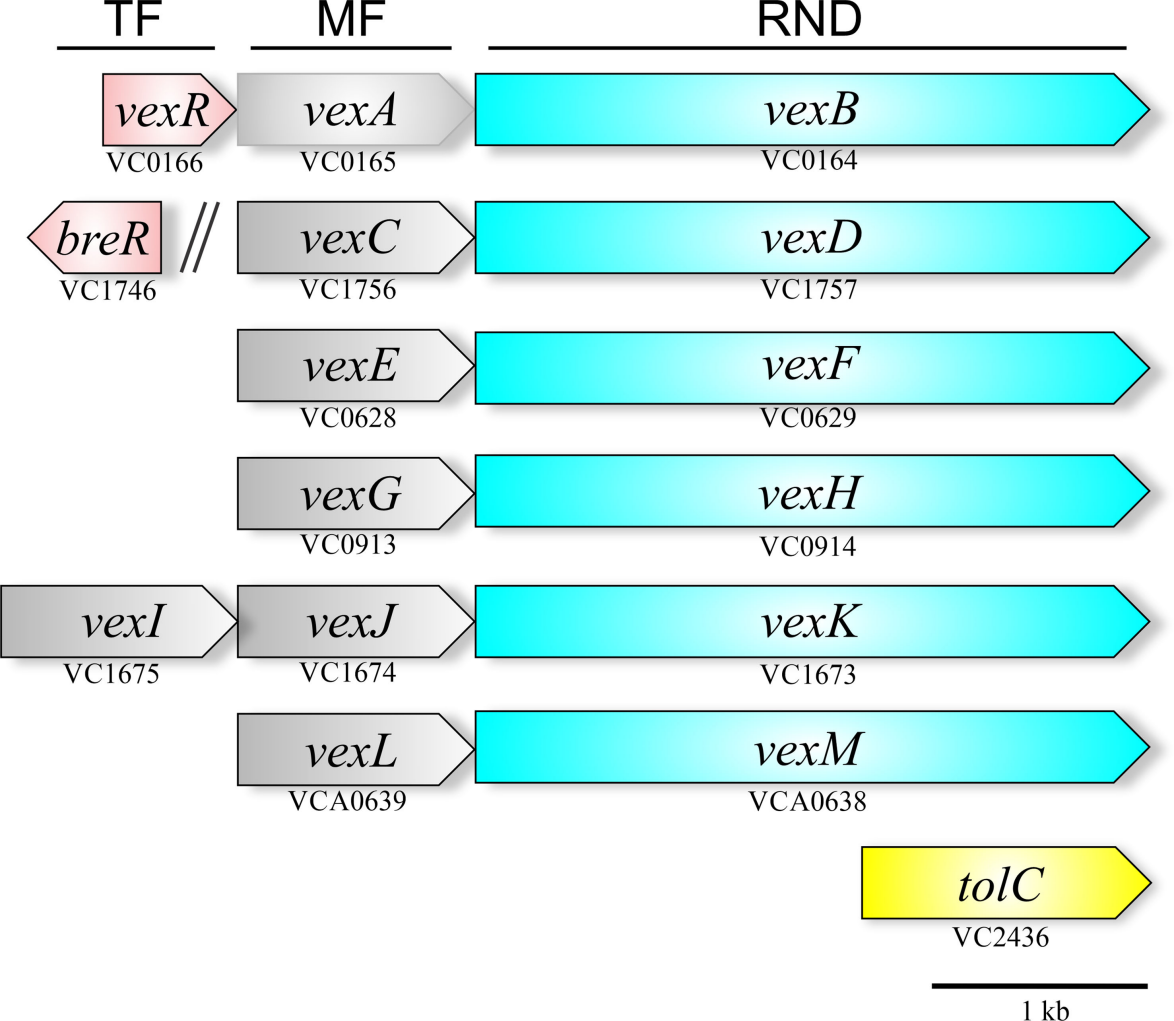
Genetic arrangement of the *V. cholerae* RND efflux operons. The six operons encode a membrane fusion protein (gray) upstream of the RND-family pump protein (blue). Five of the operons are encoded on the large chromosome with one on the small chromosome (*vexLM*). Two TetR-family regulators (red) have been linked to the RND systems, VexR, which regulates the *vexRAB* operon, and BreR, which regulates the *vexCD* operon. The outer membrane pore protein is provided by *tolC* (yellow) is encoded separately in the genome.

*V. cholerae*, like most gram-negative bacteria harbors multiple RND transporter operons. There are six RND systems encoded in its genome: VexAB, VexCD, VexEF, VexGH, VexIJK, and VexLM (Bina et al., 2006; Bina et al., 2008); five are located on the large chromosome and *vexLM* on the small chromosome. These operons display an arrangement typical of RND genes in most bacteria with the membrane fusion coding sequence located directly upstream of the RND pump ORF (**Figure 3**). The *vexIJK* operon is an exception to this rule because it includes two membrane fusion genes (i.e., *vexI* and *vexJ*); the biological significance of the two membrane fusion proteins is unknown. None of the six *V. cholerae* RND efflux operons include a gene coding an outer membrane pore. The TolC homologue appears to be a shared outer membrane pore among the six RND systems in *V. cholerae*. The *tolC* (VC2436) coding sequence is encoded as a single, self-standing gene on the large chromosome (Bina and Mekalanos, 2001). TolC’s RND pore function was inferred from experiments showing that a *tolC* deletion mutant exhibited the same antimicrobial resistance and virulence factor production phenotypes as an otherwise isogenic RND efflux pump negative strain that retains *tolC* (Bina and Mekalanos, 2001; Weng et al., 2021). VexR, a transcriptional regulator belonging to the TetR family has been reported to positively regulate the expression of the *vexRAB* operon (Bina et al., 2008; Taylor et al., 2015), while BreR, another member of the TetR family is a transcriptional regulator that represses *vexCD* expression (Cerda-Maira et al., 2008; Cerda-Maira et al., 2013).

## RND systems mediate *V. cholerae* antimicrobial resistance

RND efflux systems afford gram-negative bacteria intrinsic antimicrobial resistance while contributing to the evolution of multiple antibiotic resistant pathogens. *V. cholerae* RND systems have been linked to xenobiotic resistance, but their contributions to the evolution of multiple antibiotic resistant strains have not been investigated. RND transporters provide resistance *in vivo* against multiple toxic molecules including bile salts, detergents, and antimicrobial peptides to which *V. cholerae* is exposed to while colonizing the human small intestine (Alav et al., 2021). The contribution of RND efflux system towards *V. cholerae* antimicrobial resistance was tested experimentally by comparing a mutant lacking all six RND pump protein operons to otherwise isogenic strains encoding single or select RND pumps (Bina et al., 2006; Bina et al., 2008). VexEF was also assessed by expression of the operon in *E. coli* (Rahman et al., 2007). Deletion of all six RND transporters resulted in hypersensitivity to multiple antimicrobial compounds including antibiotics, antimicrobial peptides, bile salts, dyes and detergents (**Table 1**) (Bina et al., 2008). VexAB conferred resistance to bile acids, detergents, defensins, dyes and several antibiotics, including erythromycin, polymyxin B, penicillin, ampicillin and novobiocin demonstrating a broad-spectrum multiple drug efflux phenotype indicative of VexAB being the main contributor to *V. cholerae* intrinsic antimicrobial resistance. Furthermore, the *vexB* single deletion mutant and the RND-null strain displayed identical minimal inhibitory concentrations (MIC) of erythromycin, polymyxin B, and penicillin, attributing resistance to these antibiotics solely to the VexAB RND system (Bina et al., 2006). The remaining efflux pumps revealed comparatively limited substrate specificities that were redundant with those of VexAB. No compounds tested in these experiments were substrates for the *vexLM* operon gene products suggesting a higher extent of specificity and/or efflux of compounds not evaluated; for example, those encountered in the aquatic environment. The *vexCD* operon functioned exclusively in bile acids efflux (Bina et al., 2006; Bina et al., 2008) while VexGH was implicated in resistance to ampicillin, bile acids, novobiocin and the nonionic detergent Triton X-100 (Taylor et al., 2012). VexIJK efflux mediated resistance to novobiocin, SDS and Triton X-100 (Bina et al., 2008; Taylor et al., 2012) and a combination *vexBDHK* deletion mutant exhibited an antimicrobial susceptibility profile identical to the RND deficient mutant (Bina et al., 2008; Taylor et al., 2012). VexB, VexD, VexK, and VexH are likely the only RND pumps that contribute to *in vitro* antimicrobial resistance. Taken together, these experiments indicate that *V. cholerae* expresses multiple, functionally redundant pump systems to efflux bile acids and detergents supporting an important role for RND proteins towards adaptation during colonization of the small intestine. Redundant bile efflux mechanisms provide an adaptation that allows pathogens to overcome an intrinsic colonization barrier in the small intestine where bile acts as a natural host defense against microorganisms.

**Table 1:** Substrate specificity of the *V. cholerae* RND efflux systems.

	RND system					
	VexAB	VexCD	VexGH	VexIJK	VexEF	VexLM
**Antimicrobial peptides**	yes	no	no	no	no	no
**β-lactams**	yes	no	yes	no	no	no
**Bile acids**	yes	yes	yes	no	yes*	no
**Dyes**	yes	no	no	no	yes*	no
**Erythromycin**	yes	no	no	no	yes*	no
**Novobiocin**	yes	no	yes	yes	yes*	no
**SDS**	yes	no	no	yes	no	no
**Triton X-100**	yes	no	yes	yes	yes*	no

*Only observed when expressed in Escherichia coli.

Although VexB, VexD, VexH, and VexK efflux pumps exhibited redundant specificity for some substrates, they did not contribute equally to antimicrobial resistance. For example, all four pumps enhanced resistance to bile acids, but in the presence of VexB the loss of the other three transporters did not have an impact (Bina et al., 2008). Further, the contribution of VexH and VexK to bile resistance was only observed in a *vexBD* negative background (Bina et al., 2008; Taylor et al., 2012). These observations suggest that VexB and VexD are the major contributors to bile acid resistance *in vitro*, while the contribution of VexH and VexK are minor under laboratory experimental conditions. Transcriptomic analyses of cholera patient stool and a human volunteer study indicated that *vexAB*, *vexGH* and *vexIJK* are expressed in the human intestine (Bina et al., 2003; Lombardo et al., 2007). Taken together, these results support the notion that VexAB is the principal RND efflux system in *V. cholerae* and that VexIJK and VexGH may play a greater role *in vivo*.

Importantly, these observations may not reflect the contributions of individual RND transporters to resistance under alternate experimental conditions, including *in vivo* because RND transporter expression is regulated in response to environmental cues. Moreover, individual *V. cholerae* RND transport systems expressed episomally in *E. coli* revealed that recombinant *vexEF* led to decreased susceptibility against multiple antimicrobials (e.g. bile acids, dyes, erythromycin, novobiocin and Triton X-100), indicating that VexEF is capable of acting as a broad spectrum multiple drug efflux system (Rahman et al., 2007). In contrast, deletion of *vexF* in *V. cholerae* did not affect antimicrobial resistance for reasons that remain unclear but may related to intrinsic low expression levels (Bina et al., 2008).

## RND efflux contributes to *V. cholerae* host colonization

*V. cholerae* lacking RND efflux systems exhibits severe attenuation in infant mouse colonization which was hypothesized to result from an abundance of antimicrobial compounds in the gastrointestinal tract (Bina et al., 2008). RND-mediated efflux together with outer membrane selective permeability is hypothesized to synergistically allow bacteria to overcome the cytotoxic effect of bile acids, detergents and antimicrobial peptides commonly found in the intestine (Bina and Mekalanos, 2001; Bina et al., 2008; Weng et al., 2021). Competitive index experiments in which strains are compared pairwise for infant mouse colonization revealed that the *vexBDH*, *vexBDK* and RND null mutants could not be recovered from infant mice when administered at a 1:1 ratio with WT. All the other single and combination mutants were colonization competent comparably to WT including the *vexDHKFM* mutant with an intact *vexB*. These results confirmed that VexB can complement the loss of the other five RND efflux systems, and that the other RND transporters were redundant with VexB in this animal model (Bina et al., 2008). In contrast, *vexBDH*, *vexBDK* and *vexBDHK* mutants inoculated into mice at a 100:1 mutant to WT ratio exhibited 3.7, 4.4 and 4.1 log reductions in recovery relative to wild type (WT), respectively. Furthermore, the RND null mutant remained unrecoverable at the same inoculation ratio with WT as well (Taylor et al., 2012). This finding further supports the notion that all six RND systems contribute towards colonization.

Separate from the strictly protective physiological function which impact colonization of the small intestine by enhancing antimicrobial resistance, RND efflux evolved to tie into virulence gene expression. CT and TCP transcription in RND-efflux deficient *V. cholerae* is decreased by ~70% when the bacteria are grown in laboratory conditions that induce virulence factor expression, suggesting a regulatory link between RND-mediated efflux and expression of the ToxR regulon (Bina and Mekalanos, 2001; Bina et al., 2008; Weng et al., 2021). Interestingly, the contribution of individual RND efflux systems towards antimicrobial resistance mirrored their impact on virulence factor production. For example, a *vexBDHK* mutant expressed lower amounts of CT and TCP but the RND null mutant even less, suggesting that the VexLM and VexEF RND systems, which do not contribute towards antimicrobial resistance, do play a role in virulence factor production (Bina et al., 2008; Taylor et al., 2012). In contrast, levels of CT and TCP production of *vexBDH*, *vexBDK*, *vexBHK*, and *vexDHK* mutants did not differ significantly from WT, suggesting that the VexAB, VexCD, VexGH, and VexIJK efflux systems are redundant for CT and TCP production (Bina et al., 2008; Taylor et al., 2012). The presence of VexAB alone complemented the loss of the other RND systems with regards to virulence factor production. The observation that *vexBDH and vexBDK* mutants were attenuated for colonization, but not for CT and TCP production, led to the conclusion that the RND systems played a dual role in *V. cholerae* pathogenesis with the RND efflux systems being critical both for resistance to antimicrobial compounds present in the gastrointestinal tract and for virulence factor production thereby impacting colonization multifold. VexLM and VexEF RND contribution towards *V. cholerae* colonization remains subject of speculation as it may result from its impact on virulence factor production, enhancement of antimicrobial resistance, through some other, yet to be determined activity or any combination thereof.

## RND efflux activity influences *V. cholerae* two-component system activity

*In vitro* virulence inducing conditions (i.e., AKI conditions) consist of an artificial set of *in vitro* parameters that activate the ToxR regulon and have been shown to mimic specific aspects of the events that induce expression of virulence factors *in vivo* (Iwanaga and Yamamoto, 1985). The effects of the RND efflux systems on the *V. cholerae* transcriptome were investigated by whole genome RNA sequencing of the O1 El Tor strain N16961 grown under AKI conditions. Loss of RND-mediated efflux resulted in reprogramming of the *V. cholerae* transcriptome with >350 genes being identified as differentially expressed between the efflux null mutant and WT (Bina et al., 2018). Functional classification of differentially expressed genes revealed that most had predicted functions in cell metabolism, transport and binding, virulence and regulation. Most of the genes in the ToxR virulence regulon were downregulated, suggesting that RND-mediated efflux is required for virulence factor production and may have contributed to the *in vivo* colonization defect observed in RND efflux impaired strains. The RND efflux-dependent transcriptome was also enriched for TCS, environmentally responsive regulatory systems that consist of a membrane-linked sensor kinase that relays environmental cues to a response regulator by a phosphorelay mechanism (Mitrophanov and Groisman, 2008). The phosphorylated response regulator typically functions as a transcription factor to transduce extracellular cues into transcriptional responses. Genes associated with TCS represented 57% of the differentially expressed regulatory genes in the RND mutant, whereas TCS represent just 17% of the regulatory genes in the *V. cholerae* genome (Heidelberg et al., 2000). Given that the RND systems transport substrates from the periplasm and cytoplasmic membrane, this observation suggested a novel feedback mechanism between environmental adaptation and RND-mediated efflux where metabolites normally removed from the cell by the RND systems accumulate intracellularly and initiate feedback mechanisms by environmental sensors (e.g., one- and two-component regulatory systems) leading to the expression of adaptive responses. Further investigation between the link between efflux and adaptation are described below.

## RND efflux activity modulates adaptive responses *via* ToxR

**Figure 4:**
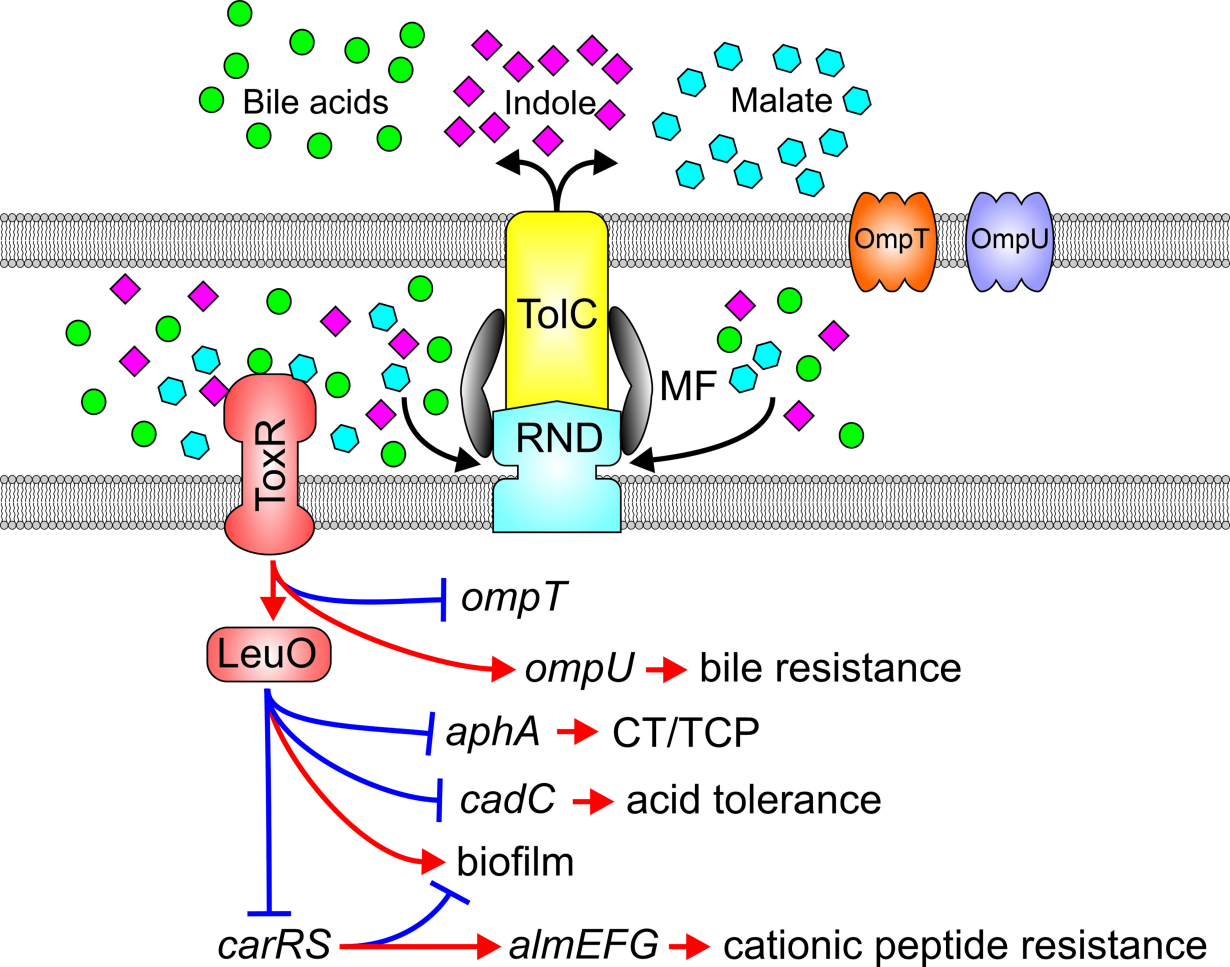
Substrates of the RND efflux systems mediate adaptive responses *via* ToxR. Impaired RND-mediated efflux results in the periplasmic accumulation of endogenous and exogenous RND substrates such as bile acids (green balls), indole (violet diamonds) or malate (blue hexagons) which engage the ToxR periplasmic domain to activate ToxR and downstream adaptive responses.

LeuO is a recently described LysR-type regulator in *V. cholerae* that regulates biofilm production and acid tolerance response (Moorthy and Watnick, 2005; Ante et al., 2015a). ToxR activates *leuO* transcription by binding to a consensus promoter site; an event that appears to occur constitutively in the absence of RND-mediated efflux, leading to elevated *leuO* transcription (Bina et al., 2018). Interestingly, ToxR’s periplasmic sensing domain is required for activation of *leuO* transcription suggestive of an efflux-dependent small molecule feedback mechanism tying ToxR to *leuO* expression. Consistent with these observations, ToxR induced *leuO* expression in the presence of multiple RND efflux substrates including bile acids, indole, and diketopiperazines, the former two being RND efflux substrates that are abundant in the human gastrointestinal tract (Bina et al., 2009; Bina and Bina, 2010; Bina et al., 2013; Ante et al., 2015b; Howard et al., 2019; Bina et al., 2021). Furthermore, the RND efflux inhibitor phenylalanine-arginine β-naphthylamide induced *leuO* expression by a mechanism dependent on the presence of the ToxR periplasmic sensing domain suggestive of a regulatory feedback circuit that inhibits efflux of the transporter’s native substrates to modulate the ToxR regulon. These findings led to the formulation of a model whereby RND systems influence adaptive responses as a direct result of endogenous and exogenous metabolite efflux from the periplasmic space where they are otherwise sensed by ToxR (**Figure 4**). In the absence of RND efflux activity, through mutation or inhibition by chemicals, metabolites normally expelled accumulate in the periplasmic space and activate ToxR by interacting with its periplasmic sensing domain. ToxR, independent of TcpP then induces expression of *leuO*, which leads to virulence repression and altered cell physiology (Bina et al., 2018). Metabolite accumulation may also result from high cell density, consistent with the observation that LeuO is expressed in stationary phase of growth. Overexpression of *leuO* negatively regulates *aphA* thereby repressing production of CT and TCP. Given LeuO’s natural expression during stationary phase of growth when cell densities are high, it is tempting to speculate that this novel ToxR regulon branch is involved in *V. cholerae*’s late infection phenotype.

The effects of efflux on ToxR and LeuO signaling pathways extend beyond virulence to additional adaptive responses that are likely important to the *V. cholerae* pathogenic lifecycle including host adaptation or dissemination or transmission. ToxR and LeuO play additional roles in bile resistance independently of RND transporters thereby further protecting *V. cholerae* from the toxic effect of bile in the intestine. ToxR and LeuO also mediate acid tolerance *via* LeuO-mediated repression of the tightly regulated *cadC-cadBA* system critical for maintaining pH homeostasis and cell survival in acidic milieus (Ante et al., 2015a). Furthermore, ToxR regulates antimicrobial peptide resistance *via* LeuO-dependent repression of the *carRS* TCS (Bina et al., 2016). CarRS positively regulates the *almEFG* operon which modifies lipopolysaccharides with glycine and diglycine to mediate high-level cationic antimicrobial resistance in *V. cholerae* (Bilecen and Yildiz, 2009; Herrera et al., 2014; Bilecen et al., 2015). Lastly, ToxR and LeuO also contribute to biofilm production, which is critical for hyperinfectivity and environmental survival, but the mechanism by which this occurs has not been fully resolved (Moorthy and Watnick, 2005; Bilecen et al., 2015; Kazi et al., 2016).

## Reciprocal relationship between RND-mediated efflux and the Cpx response

The Cpx system is widespread among Gammaproteobacteria where it functions to mitigate envelope stress resulting from environmental perturbations such as high salinity, magnesium ion concentration, oxidative stress, low iron, and proteins containing aberrant disulfide bonds (Hews et al., 2019). CpxA is a membrane-associated histidine kinase sensor; when stimulated this sensor phosphorylates the CpxR response regulator which then binds to a conserved consensus sequence in the promoter of target genes to modulate adaptive responses (Taylor et al., 2014). CpxR also regulates expression of *cpxP*, which is divergently expressed from the *cpxAB* operon. CpxP is a periplasmic protein thought to interact with the periplasmic domain of CpxA to modulate its activity (Hews et al., 2019). Initial studies revealed that *tolC* deletion in *V. cholerae* activated the Cpx system, suggesting a possible link between RND-mediated efflux and the Cpx system (Slamti and Waldor, 2009). Consistent with this finding the Cpx system was upregulated in RND-deficient *V. cholerae* (Bina et al., 2018). Targeted studies revealed that inactivation of the VexB or VexH RND transporters activated the Cpx system (Kunkle et al., 2017). The link between the RND efflux and the Cpx system extended beyond efflux-dependent activation of the Cpx system, as *vexRAB*, *vexGH* and *tolC* were identified as members of the Cpx regulon and positively regulated by CpxR, suggesting that the VexAB and VexGH RND transporters are components of the *V. cholerae* Cpx response intended to mitigate extracytoplasmic stress (Taylor et al., 2014; Acosta et al., 2015b; Kunkle et al., 2017).

**Figure 5:**
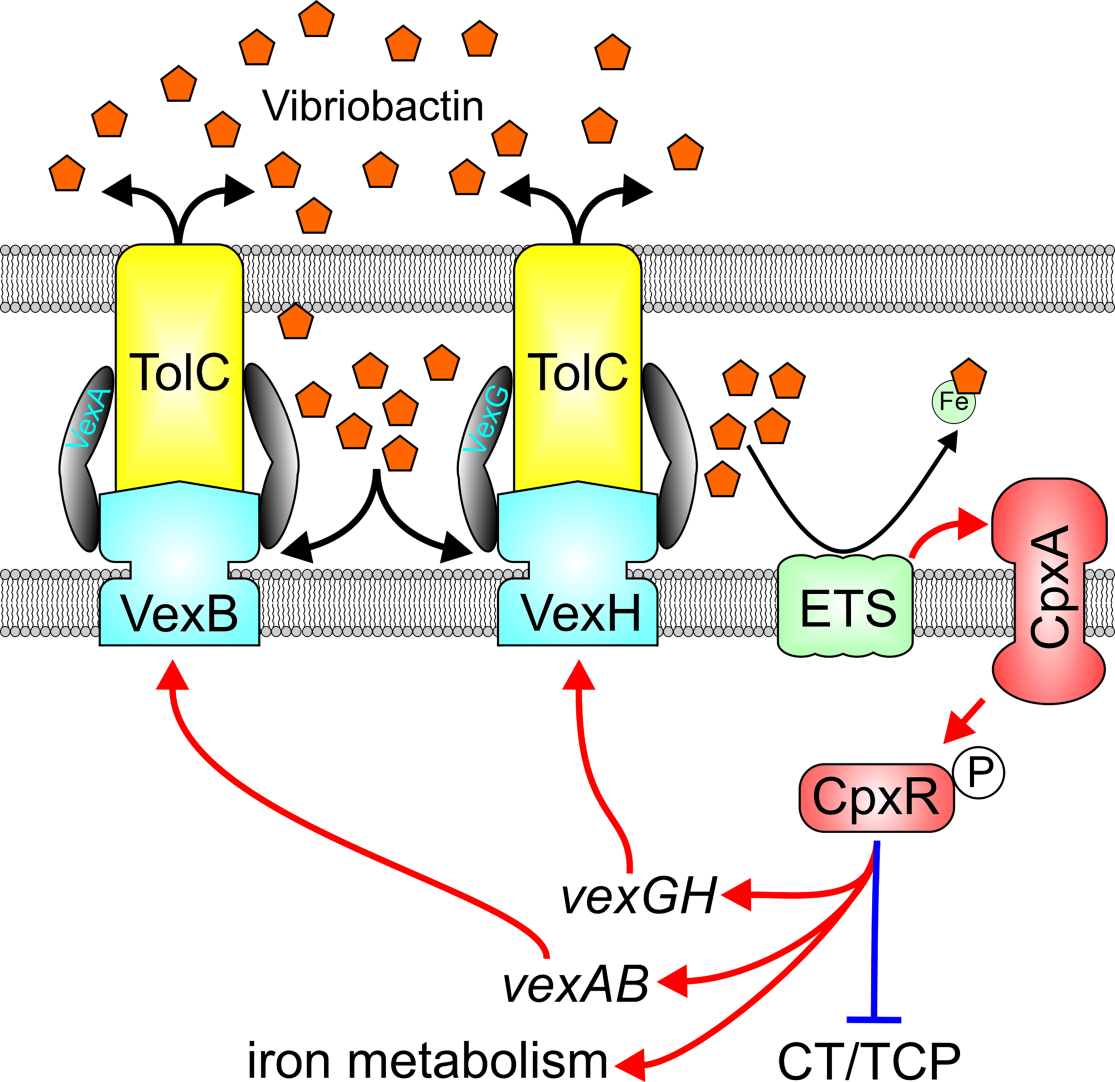
Reciprocal relationship between the VexAB and VexGH RND systems and the Cpx system. VexAB and VexGH contribute to efflux of the siderophore vibriobactin, and impaired efflux results in vibriobactin accumulation in the periplasm which titrates iron from iron-containing components of the electron transport system (ETS) resulting in protein misfolding and activation of the cpx system. Cpx system activation results in induction of *vexAB*, *vexGH* and iron homeostasis genes while repressing virulence gene expression.

Constitutive activation of the Cpx system in RND-impaired cells suggests that periplasmic accumulation of metabolites normally expelled from *V. cholerae* periplasm by the VexB and VexH RND transporters are likely responsible for Cpx activation. A Cpx-inducing metabolite was identified from a transposon library screened for suppressors of the Cpx system in RND efflux negative *V. cholerae* (Kunkle et al., 2017). Transposon insertions into succinate dehydrogenase coding sequence or genes involved in synthesis of vibriobactin, an iron siderophore, suppressed expression of the Cpx system. This suggested that the RND transporters are likely involved in vibriobactin efflux, a finding confirmed with directed studies showing that VexAB and VexGH mediate siderophore’s secretion (**Figure 5**) (Kunkle et al., 2017). This led to the formulation of a model where in the absence of VexAB or VexGH, vibriobactin accumulates in the periplasm, titrated iron from iron-containing members of the electron transport chain such as succinate dehydrogenase either leads to formation of aberrant disulfide bonds or misfolded membrane proteins, which in turn stimulate activation of the Cpx system. Consistent with these observations, the Cpx system functions as an adaptation to iron-limiting conditions (Acosta et al., 2015b) *V. cholerae* may encounter *in vivo*. Interestingly, ectopic overexpression of CpxR was shown to repress *V. cholerae* virulence factor production (Acosta et al., 2015a), suggesting another potential link between the Cpx system, RND-mediated efflux and virulence.

## The EnvZ-OmpR regulon is integrated into the ToxR virulence regulon through RND efflux activity

**Figure 6:**
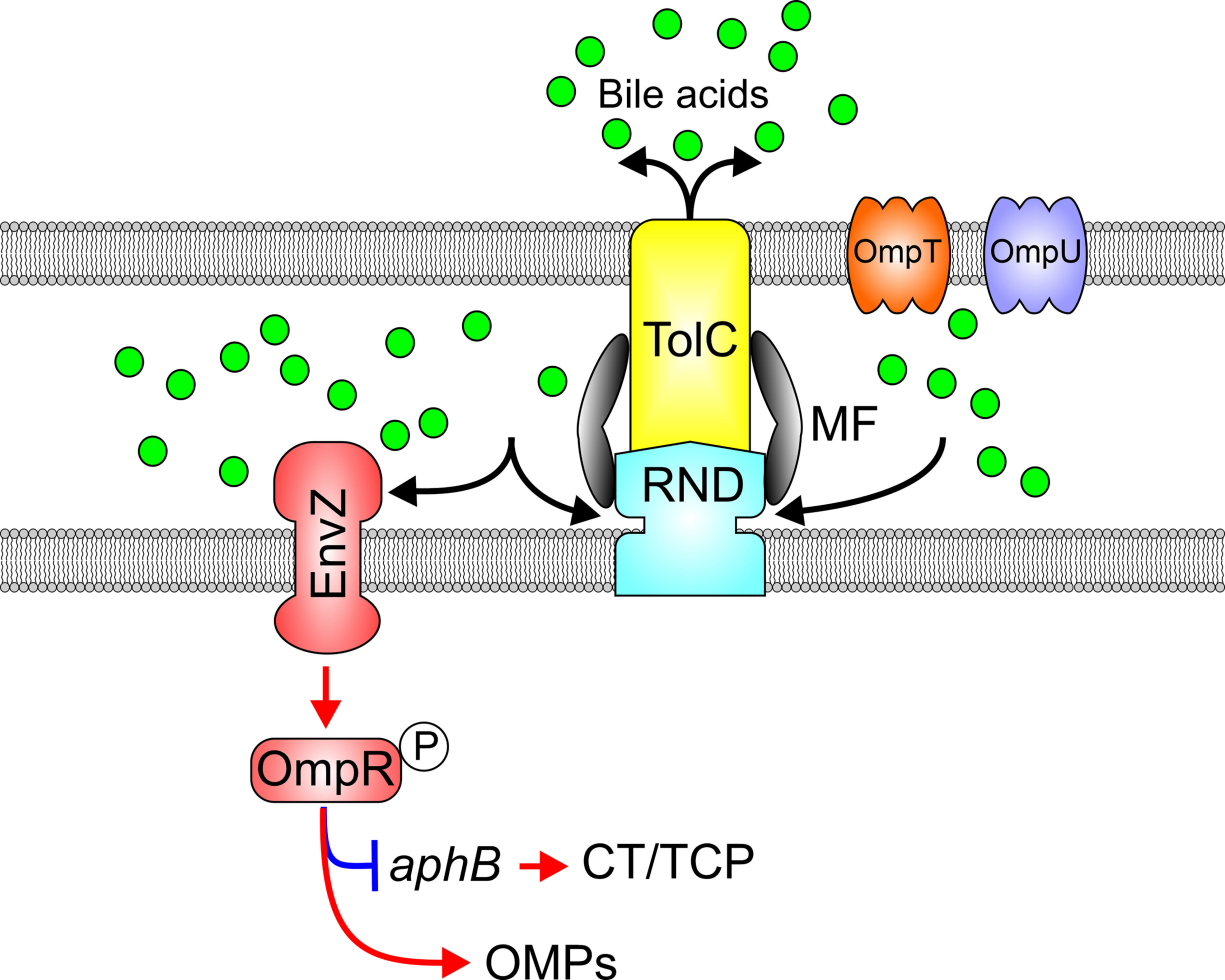
RND efflux influences the activation state of the EnvZ-OmpR TCS *via* efflux of bile acids. Bile acids (green balls) and other membrane intercalating agents induce EnvZ kinase activity and phosphorylation of OmpR which in turn modulates outer membrane porin expression and represses virulence factor production by inhibiting *aphB* transcription.

The EnvZ-OmpR TCS is activated in response to high osmolarity or low pH among Enterobacteriaceae where EnvZ is a membrane associated histidine kinase sensor and OmpR is the DNA binding response regulator (Sarma and Reeves, 1977; Hall and Silhavy, 1981; Slauch et al., 1988). OmpR has also been linked to virulence (Bernardini et al., 1990; Lee et al., 2000; Schwan, 2009; Reboul et al., 2014; Tipton and Rather, 2017; Lin et al., 2018; Silva et al., 2018) and acid tolerance (Heyde and Portalier, 1987; Bang et al., 2000; Hu et al., 2009; Gao et al., 2011; Stincone et al., 2011; Quinn et al., 2012) in gram-negative bacteria. The *V. cholerae envZ-ompR* operon was found to be upregulated in the absence of RND mediated efflux, suggesting that it may contribute to the virulence repression phenotype observed in this background (Bina et al., 2008; Bina et al., 2018). Deletion of *ompR* in an RND-negative *V. cholerae* background raised virulence factor production (Kunkle et al., 2019). Conversely, *ompR* overexpression in WT cells repressed CT and TCP biosynthesis confirming that OmpR acts as a virulence repressor (Kunkle et al., 2020a). Transcriptomic studies revealed that OmpR controlled expression of a large regulon that included outer membrane proteins, virulence factors and genes involved in acid tolerance (Kunkle et al., 2020b). Interestingly, the *V. cholerae* EnvZ-OmpR system did not respond to canonical inductive stimuli (i.e. high salt or low pH) typical of Enterobacteriaceae, but instead was induced in response to membrane intercalating agents including bile salts, detergent-like molecules, and alkaline pH (Slamti and Waldor, 2009; Kunkle et al., 2019). Interestingly, AKI growth media passed through a C18 reverse phase chromatography column revealed the presence of multiple bile acids acting as activating factors responsible for *ompR* induction in RND negative *V. cholerae* (Kunkle et al., 2019). Induction of *ompR* in RND deficient *V. cholerae* likely resulted from intracellular accumulation of bile salts in the RND-null mutant (**Figure 6**). The link between RND efflux and OmpR-dependent virulence repression was linked to OmpR directly repressing *aphB* transcription, thus, revealing a new regulatory arm of the ToxR regulon (Kunkle et al., 2019). Integration of OmpR into the ToxR regulon represents a third mechanism by which RND-mediated efflux contributes virulence. Subsequent experiments revealed that the EnvZ-OmpR system is also activated in response to alkaline pH, and shown to regulate multiple acid-tolerance genes through a phenotype that appeared to be independent of the RND efflux but could have contributed to decreased colonization fitness of an *ompR* mutant in infant mice (Kunkle et al., 2020b).

## Conclusions

The lifecycle of pathogenic *V. cholerae* relies on a continuum of adaptations required to transition between disparate environments within the aquatic ecosystem and survival through the human gastrointestinal tract, from traversing the gastric acid barrier of the stomach to colonization in the duodenum, jejunum and ileum [reviewed in (Kaper et al., 1995)]. Each of these anatomical sites presents distinct environmental cues produced from the host and resident flora, and within each site local environmental differences between the lumen and the epithelium are dramatic. The ability of *V. cholerae* to colonize and replicate in each of these respective sites requires rapid transcriptional adaptation in response to environmental cues including exogenous and endogenous cues that are substrates of RND efflux systems such as organic acids, antimicrobial peptides, bile salts, fatty acids, and indole (Bina et al., 2013; Taylor et al., 2014; Ante et al., 2015b; Bina et al., 2018; Howard et al., 2019; Kunkle et al., 2019; Kunkle et al., 2020b; Bina et al., 2021). These compounds are concentration-dependent effectors and their influence on adaptive responses is directly linked to RND-mediated efflux. The intracellular accumulation of each of these effectors is therefore linked to cell metabolism and the maintenance of the proton motive force which is the energy source for RND-mediated efflux. Thus as *V. cholerae* enters stationary phase, a time when a stringent response is induced and the proton motive force begins to dissipate, these molecules may accumulate intracellularly and induce adaptive responses that are necessary for transmission and dissemination (Bina et al., 2018). Further, as endogenous metabolites are expelled from *V. cholerae* by the RND efflux systems, competitive inhibition of efflux by exogenous compounds (e.g., bile acids in the small intestine) may further drive adaptive responses contributing to colonization and persistence. Finally, concentration gradients of molecules in the host GI tract may have a profound impact on the pathogenic success of *V. cholerae in vivo* (Duane et al., 1986). For example, bile acids are important signaling molecules that affect numerous virulence-associated phenotypes (Provenzano and Klose, 2000; Provenzano et al., 2000; Wibbenmeyer et al., 2002; Cerda-Maira et al., 2008; Yang et al., 2013; Ante et al., 2015b; Midgett et al., 2017; Bina et al., 2021) and the bile-acid gradient across the mucous layer covering the intestinal epithelium may drive differential phenotypic evolution of *V. cholerae* localized to the epithelial surface compared to the lumen.

The broad specificity of RND transporters is critical for their roles in the evolution of multiple drug resistant pathogens and studies to develop efflux inhibitors to reverse efflux-mediated resistance are ongoing (Compagne et al., 2023). However, the RND systems likely evolved to fulfil physiological functions in bacteria, suggesting a paradigm where the contribution RND efflux systems to antimicrobial resistance is secondary to their native functions in maintaining cell homeostasis. This conclusion is supported by *V. cholerae* studies described herein showing that the loss of RND transport systems have widespread effects on multiple physiological functions including iron homeostasis, acid tolerance, biofilm production, and membrane remodeling. Similar findings have been reported in multiple other pathogens suggesting that the function of the RND systems in bacterial physiology is conserved across gram-negative pathogens [reviewed by (Alvarez-Ortega et al., 2013; Colclough et al., 2020)]. As many of these RND-dependent phenotypes are important for pathogenesis, the use of RND inhibitors represents a novel avenue for the development of antivirulence therapeutics that specifically impact physiology and adaptation rather than just antimicrobial resistance. Targeting bacterial physiology provides an alternative therapeutic strategy to treat infections that has the potential to circumvent primary and secondary drug resistance mechanisms. While there have been limited efforts in this approach, experimental results suggest it is feasible and merits further exploration.

## Author contributions

All authors listed have made a substantial, direct, and intellectual contribution to the work, and approved it for publication.
